# Biological Invasions of Geminiviruses: Case Study of TYLCV and *Bemisia tabaci* in Reunion Island

**DOI:** 10.3390/v4123665

**Published:** 2012-12-12

**Authors:** Frédéric Péréfarres, Magali Thierry, Nathalie Becker, Pierre Lefeuvre, Bernard Reynaud, Hélène Delatte, Jean-Michel Lett

**Affiliations:** 1 CIRAD, UMR PVBMT, Pôle de Protection des Plantes, 97410 Saint-Pierre, Ile de La Réunion, France; E-Mails: frederic.perefarres@cirad.fr (F.P.); magalithierry@wanadoo.fr (M.T.); pierre.lefeuvre@cirad.fr (P.L.); bernard.reynaud@cirad.fr (B.R.); helene.delatte@cirad.fr (H.D.); lett@cirad.fr (J.-M.L.); 2 Université de La Réunion, UMR PVBMT, Pôle de Protection des Plantes, 97410 Saint-Pierre, Ile de La Réunion, France; 3 Muséum National d’Histoire Naturelle, Département Systématique et Evolution, USM 601, CNRS UMR 5202 Origine, Structure et Evolution de la Biodiversité, 57 rue Cuvier, CP 50, 75005 Paris, France; E-Mail: becker@mnhn.fr

**Keywords:** biological invasion, *Begomovirus*, *Tomato yellow leaf curl virus*, *Bemisia tabaci*, endosymbionts, competition

## Abstract

In the last 20 years, molecular ecology approaches have proven to be extremely useful to identify and assess factors associated with viral emerging diseases, particularly in economically and socially important tropical crops such as maize (maize streak disease) and cassava (cassava mosaic disease). Molecular ecology approaches were applied in Reunion Island to analyze the epidemic of tomato yellow leaf curl disease, which has been affecting the island since the end of the 1990s. Before the invasive biotype B (currently known as Middle East-Asia Minor 1 cryptic species) of *Bemisia tabaci* spread across the world, Reunion Island (South West Indian Ocean) only hosted an indigenous biotype of *B. tabaci*, Ms (currently known as Indian Ocean cryptic species). Wild hybrids between invasive and indigenous species were subsequently characterized over multiple generations. Endosymbiont analysis of the hybrid population indicated that matings were non-random. Similarly, while no indigenous begomoviruses have ever been reported on Reunion Island, the two main strains of one of the most damaging and emerging plant viruses in the world, the Mild and Israel strains of the *Tomato yellow leaf curl virus* (TYLCV-Mld and TYLCV-IL), were introduced in 1997 and 2004 respectively. While these introductions extensively modified the agricultural landscape of Reunion Island, they also provided an invaluable opportunity to study the ecological and genetic mechanisms involved in biological invasion and competition.

## 1. Introduction

Biological invasions are a major agent of global change. They are often linked to emerging diseases [1] and often have negative effects on biodiversity and on the economy [2]. This is particularly true in insular ecosystems where small isolated populations allow rapid evolution and where the negative effects of invasion are more pronounced [3,4]. This makes the study of species introduced in insular ecosystems particularly appealing when it comes to dealing with agricultural issues. Humans have deliberately or accidentally introduced thousands of non-indigenous animal and plant species on such islands [2]. To become invasive in a new area, a species must successfully complete a series of fundamental steps: introduction, establishment, increase in number and geographical spread [5].

In this review, we focus on biological invasions in agricultural settings and on the worldwide emergence of the whitefly *Bemisia tabaci* and begomoviruses, most notably *Tomato yellow leaf curl virus* (TYLCV). Over the past 30 years, whiteflies and begomoviruses have become serious threats to the cultivation of a variety of vegetable crops of great importance in different parts of the world but especially in the tropics and sub-tropics. After a brief review of historically recent examples of successful biological invasions of geminiviruses in agricultural settings and their respective drivers, we trace the worldwide dissemination of invasive biotypes of *B. tabaci* and TYLCV. We also describe the fundamental role of endosymbionts in their whitefly host ecology and evolution through a wide range of effects, and their likely effect on virus acquisition and long-term retention. Finally, we describe what took place in Reunion Island, whose invasion by *B. tabaci* and TYLCV strains profoundly modified the agricultural landscape.

## 2. Discussion

### 2.1. Emergence of Geminivirus Diseases in Agricultural Settings

Biological invasions are historically tightly linked with the spread of plants that human have domesticated for food, fibers, and medicinal and ornamental purposes [6,7]. Major migrations of modern man started over 8000 years ago and led to the propagation of cultivated plants and livestock. This process was so intensive that today only a small number cultivated plants (maize, rice, wheat) and animal species (domestic chickens, cattle) are found throughout the world and provide more than 98% of the world’s food supply [8].

By moving cultivated plants away from their centers of domestication to distant regions or other continents, human populations have been responsible for new encounters between plants and pest species such as viruses, fungi, bacteria, phytoplasmas and nematodes [9,10]. While pest epidemics are generally considered to be rare in indigenous plant communities, modern agricultural practices, such as monocultures, favor frequent and damaging pest epidemics [11]. In addition, by decreasing the magnitude of the evolutionary response necessary for a pest to adapt to the conditions in new and remote territories, the worldwide homogenization of the agricultural landscape has improved the success of invasion. Among emerging plant diseases, viruses are the main pests and account for about the half of emerging diseases reviewed in Anderson *et al.* [12]. These epidemics may cause serious crop losses which have dramatic social and economic consequences when they affect staple crops (e.g., maize, rice, wheat and potato) or cash crops (e.g., citrus, cacao, coffee and banana) [1,12]. While viral emergences are generally multi-factorial and are still poorly understood, it is nevertheless possible to detect general trends driving plant viral emergences. The introduction of plant viruses to new regions is the most important driver of plant viral emergences followed by changes in the vector population, the effect of recombination and/or mutation, the weather, and finally changes in farming practices [1,12]. Because of the wide variety of factors involved, interdisciplinary approaches are needed to study the emergence of plant viruses. In this context, molecular ecology (a recent branch of biological sciences which combines biology, ecology, epidemiology, molecular evolution and genetics) has emerged and is now widely used to analyze the complex patterns of virus emergences. Among the economically and socially most important viral emerging plant diseases transmitted by arthropod vectors, maize streak disease (MSD) and cassava mosaic disease (CMD) have helped highlight the wide range of factors involved in a successful viral emergence at a continental scale. These two diseases will be used in this review to illustrate viral emerging diseases and their respective drivers.

#### 2.1.1. Maize Streak Disease in Africa

Maize streak disease is a major constraint on maize production in all temperate and tropical regions in sub-Saharan Africa [13]. It has a complex, poorly understood epidemiology, with periodic outbreaks that devastate the maize yields of small- and medium-scale farmers [14]. Maize streak virus strain A (MSV-A, genus *Mastrevirus*, family *Geminiviridae*) is the causal agent of maize streak disease in Africa and surrounding islands. Whereas the majority of MSV strains (classified as MSV-A through -K) are apparently adapted to infecting wild grass species, only MSV-A isolates are adapted to infecting maize [14]. The contribution of recombination to the genetic diversification of MSV has, like for other geminiviruses, been widely demonstrated [15,16]. A recombination event between ancestral MSV-B and MSV-G/F variants is credited with having generated a maize-adapted MSV-A prototype, possibly within 20 years of the first credible reports of MSD in southern Africa in the 1870s [17]. Several observations suggest that this recombination event was the key to the emergence of MSV-A as a viral pathogen of maize. After its initial emergence and subsequent spread throughout the continent, five distinct MSV-A subtypes (MSV-A1, -A2, -A3, -A4, and -A6) evolved, and today, each has a different well-defined geographical range in sub-Saharan Africa [18]. Although it is evident that over the last century, the movement of certain MSV-A subtypes across Africa has been far less constrained than the movements of either other MSV strains or related African streak virus species, it is also evident that the different MSV-A subtypes are not all equally mobile. For example, whereas the MSV-A1 subtype is found throughout Africa, the MSV-A2, -A3, -A4, and -A6 subtypes have been found only in West Africa, East Africa, southern Africa, and Reunion Island, respectively. Using model-based phylogeographic analyses of hundreds of fully sequenced MSV-A isolates, a plausible history of MSV-A movements over the past 150 years was reconstructed [19]. Besides confirming southern Africa as the probable origin of MSV-A, an average rate of movement over the past century of 32.5 km per year was also inferred [19]. More than just the result of the evolutionary dynamics of the MSV-A strain, the epidemiology of MSD in Africa and its erratic nature is certainly the result of the complex interactions between multiple environmental and ecological factors [13]. The epidemic spread of MSD is usually attributed to the convergence of factors such as: (1) climatic conditions (temperature, rainfall, relative humidity), which increase the density of leafhopper populations, and environmental factors, which drive the long distance movement of leafhoppers; (2) the presence within leafhopper populations of a high percentage of MSV transmitters; (3) the population density of both MSV-A and leafhopper populations in reservoirs of wild grasses; (4) cropping practices such as the staggered growing seasons of maize (for reviews see [13,14]). The complex interplay of all these factors mean MSD epidemics can cause insignificant to dramatic crop losses, which are extremely difficult to predict.

#### 2.1.2. The African Cassava Mosaic Disease Pandemic

Cassava is a major staple food crop in tropical developing countries and is becoming a significant cash crop option. Cassava mosaic disease is the most important biotic constraint to production and is caused by a complex of African begomovirus species (family *Geminiviridae*) [20,21]. After its first description in Tanzania in 1894, CMD was reported in most of the cassava-growing regions in East and West Africa between the 1920s and the 1930s [22]. Plants affected by CMD are characterized not only by yellowing and curling of the leaves but also a serious reduction in yield. In the early 1990s, a severe epidemic began in Uganda and spread throughout East and Central Africa causing dramatic crop losses which required international intervention to prevent widespread famine [23]. The pandemic was also described as advancing along a ‘front’ which, in Uganda, was estimated to be moving 20 to 50 km per year [24]. Key characteristics of the pandemic were high incidences of severe symptoms of CMD, consistent mixed infection of the recombinant Uganda strain (also called Uganda variant) of East African mosaic virus (EACMV-UG) with African cassava mosaic virus (ACMV) [25], the synergism between these viruses [23,26], and rapid transmission by super-abundant *B. tabaci* populations [27]. The molecular mechanisms behind the synergism between ACMV and EACMV-like viruses include the combined action of two geminiviral suppressors of gene silencing [28]. While a clear association has been demonstrated between the CMD pandemic and the occurrence of EACMV-UG [23], the presence of EACMV-UG in countries not affected by the pandemic has also been reported in South Africa [29], Zimbabwe [29] or Burkina Faso [30]. From being a key driver of the CMD across Africa, these reports identify the Uganda variant as a contributory rather than a determining factor in the pandemic.

Although the causes of the CMD pandemic in Africa are not fully understood, it is clear that the synergism between viruses combined with the abundance of cassava whiteflies (newly adapted or introduced) has been crucial in the rapid development of CMD at a continental scale. 

### 2.2. The Worldwide Emergence of a Whitefly Pest and Plant Virus Vector

#### 2.2.1. The *Bemisia Tabaci* Species Complex

Rather than simply being a vector of CMD viruses, *Bemisia tabaci* (Gennadius, order Hemiptera, family Aleyrodidae) is the exclusive vector of the whole *Begomovirus* genus. This viral genus has recently been so successful that it has become the most diverse plant virus in terms of described species [31]. As a more exhaustive view of the *B. tabaci* species has been obtained, it has become difficult to determine whether begomoviruses are an intrinsically successful viral species or if this success is partially or mostly attributable to the success of *B. tabaci*.

*B. tabaci* belongs to a complex of genetic species formerly referred as biotypes [32]. Whereas, until recently, few biotypes had been described, it has become evident that a wide range of biotypes exists and that some are particularly invasive. Albeit morphologically indistinguishable, genetic and behavioural differences (isoenzyme profiling, barcoding based on conserved genes, life history traits) have been used to identify the biotypes, and up to 28 “putative” cryptic species are now suggested to belong to the *B. tabaci* species complex [32,33,34,35].

Although *B. tabaci* has a pan-tropical origin, these insects can be found in all warmer parts of the world as well as in greenhouses in temperate areas [33,36]. *B. tabaci* is now regarded as one of the world’s top 100 invasive species (Global Invasive Species Database; [37]). In addition, it is one of the most damaging pests in open fields or protected cropping production worldwide [38]. As well as virus transmission (begomovirus but also criniviruses, carlaviruses, ipomoviruses and torradoviruses; [31]), *B. tabaci* also damages crops directly by feeding on phloem, excreting honeydew and causing phytotoxic disorders [39,40].

#### 2.2.2. Shifts in Whitefly Populations

Two members of this complex, Middle East-Asia Minor 1 (MEAM1, commonly known as B biotype) and Mediterranean (MED, commonly known as Q biotype) have now progressed well beyond their home ranges mainly as a consequence of trade in ornamental plant species [41,42]. MEAM1 first invaded southwestern USA in the late 1980s, and subsequently became a serious outbreak pest. It probably originates from the Middle East-Asia Minor (including Iran, Israel, Jordan, Kuwait, Pakistan, Saudi Arabia, Syria, United Arab Republic, and Yemen) and has spread to about 50 other countries in the world [32].

MED has become a major threat to crops due to its recent worldwide expansion (around 10 years after MEAM1), its ability to reach high population densities [43] and its resistance to different insecticides [44,45]. MED, thought to be originally restricted to the Iberian Peninsula, has recently been observed in the Mediterranean Basin [45,46,47], sub-Saharan Africa [33,48,49] and in non-Mediterranean countries including China [50], Japan [51], Mexico and the USA [52]. Its probable origins are both eastern and western Mediterranean regions [32]. The eastern region is probably the source of the Mediterranean haplotypes found in the USA (Florida and to a lesser extent, California); the western region is probably the major source of the other invaded regions.

Following their introduction into new areas, displacement of native species of *B. tabaci* by the invasive MEAM1 or MED has been demonstrated throughout the world. For instance, the rapid displacement observed of Australia cryptic species (formerly AN biotype) and Asia II3 (formerly ZHJ1 biotype) by MEAM1 in Australia and China respectively occurred through competition and asymmetric reproductive interference. During these invasion and displacement processes, there appears to have been an increased frequency of copulation leading to increased production of female progeny among the invader (MEAM1 biotype), as well as a reduced copulation and female production in the native populations (Australia and Asia II3) [53]. Later, MEAM1 was also found to be completely displaced by MED in China [54]. However, in a similar invasion in Israel, where MEAM1 is native and MED is invasive, the two species coexist [47,55] as a result of niche partitioning and stochastic processes across landscapes [56].

Because biological and genetic characteristic of MED and MEAM1 are rather different, agricultural consequences and control strategies will depend on which of the two invasive species is present. MEAM1 appears to have higher fecundity and be more competitive than MED [57], and MED appears to present a high degree of resistance to certain insecticides [44,45]. They also differ in their hostrange [58,59] and mating behavior [60,61]. Studies on genetic divergence of this whitefly species complex traditionally used only a few genes (*Cytochrome Oxydase I*, *Intergenic transcribed Spacer I* and *16S* ribosomal DNA) but recent studies based notably on transcriptomic approaches have provided new insights into genetic differences between MEAM1 and MED, notably on specific sets of genes involved in biological invasion, host adaptation, and insecticide resistance [62,63]. Indeed, as areas invaded with pests are commonly treated with several pesticides, pesticide resistance of introduced whiteflies is probably the main prerequisite for their settlement and spread [64] and some “genetic combination” linked to insecticide resistance may be a key factor in invasion success [55].

#### 2.2.3. Whitefly Population Differentiation Based on Endosymbionts

Another interesting feature of the arthropod lifestyle is their association with endosymbionts, which has greatly contributed to the evolutionary success of arthropods [65]. Heritable endosymbionts can be classified as being either obligate or facultative for the host. In contrast to obligate endosymbionts, facultative endosymbionts are not essential for host development or reproduction. *B. tabaci* harbours an obligate endosymbiont *Portiera aleyrodidarum* and can harbour up to seven facultative endosymbionts (*Cardinium*, *Arsenophonus*, *Rickettsia*, *Hamiltonella*, *Wolbachia, Fristchea*) [66,67,68,69]. Different endosymbiotic combinations have been described in the field, mainly according to former biotypes, suggesting that endosymbionts play a role in the biology of *B. tabaci* and in its differentiation into biotypes [48,70,71,72].

In the last decade, it has become clear that endosymbionts play a fundamental role in their host ecology and evolution through a wide range of effects [73,74,75], which include manipulating host reproduction [76,77,78], as well as increasing host survival or fecundity [79,80,81]. In *B. tabaci*, a role in manipulating reproduction between cryptic species has been suggested [72]. Recent studies have also pointed out that some endosymbionts can establish insecticide resistance in pest insects by detoxifying chemical agents [82]. In *B. tabaci* the link between endosymbionts and insecticides has been investigated: a high density of *Rickettsia* or *Wolbachia* was shown to be correlated with increased susceptibility, depending on the family of the insecticide compound concerned [83,84].

While these endosymbiont associations may form long-term evolutionary relationships with their arthropod hosts, they can also be dynamic, entering and leaving populations in spans of a few years to thousands of years [85,86]. For instance, Himler *et al.* [75] recently observed the rapid spread of the facultative endosymbiont *Rickettsia* sp. nr. *bellii* in the population of *B. tabaci* MEAM1 in the United States. Horizontal transmission routes, via parasitoids or plants, have recently been described for *Rickettsia* and MEAM1; these routes are possibly involved in this rapid spread [87,88]. Compared to uninfected populations, *Rickettsia*-infected populations performed better with an increase in offspring, better rates of survival until adulthood, faster development and an increased female: male sex ratio in offspring. The sex-ratio bias and performance benefits associated with *Rickettsia-*infections were sufficient to explain the sweep of *Rickettsia* in the fields across the southwestern United States [75]. Interestingly, similar differences in performance were observed between MEAM1 (harboring a greater than 90% prevalence of *Rickettsia*) and Indian Ocean species (harboring only 3% prevalence of *Rickettsia*) [72]. Moreover, the association of *Rickettsia* with increased tolerance to heat shock (40 °C) in MEAM1 [89] may explain the better performances of *Rickettsia*-harboring MEAM1 in tropical regions such as Reunion Island.

#### 2.2.4. Involvement of Endosymbionts in the Survival of Begomoviruses in their Insect Vector

Endosymbionts are likely involved in the circulative and persistent transmission of begomoviruses [90]. A chaperonin GroEL homologue (hereafter named GroEL) produced by endosymbionts is required. Immunolocalization and functional studies suggest that GroEL interacts specifically with virus coat protein (CP, [91,92]), notably to protect them from degradation in the haemolymph [90,93,94,95,96,97,98].

The endosymbionts producing the GroEL responsible for CP binding were previously believed to be secondary [93]. In *B. tabaci*, GroELs have been described for a range of secondary endosymbionts: *Hamiltonella* [91], *Arsenophonus* [92], *Rickettsia* [91], and the primary symbiont *Portiera* [91]. Subsequent comparisons of TYLCV transmission by MEAM1 and MED species (harboring different symbiotypes) indicated that *Hamiltonella*, but not *Rickettsia* or *Arsenophonus*, may be the main actors during transmission [91]. However, in Reunion Island, previous comparisons between Indian Ocean (devoid of *Hamiltonella*, but harboring *Arsenophonus* and *Cardinium*) and MEAM1 (78% prevalence for *Hamiltonella*, 91% for *Rickettsia*) revealed no significant differences in TYLCV transmission [72,99]. Therefore, interaction of GroEL with the coat protein appears to be necessary but not sufficient to explain the differences observed in the efficiency of virus transmission by the insect vectors [94]. Last but not least, a rich microbial community was reported in a recent transcriptome analysis of *B. tabaci*: a total of 17,766 bacterial unigenes were classified into 322 genera [100]. Among the NCBI-nr best blast hits in the transcriptome of *B. tabaci,* GroELs closely related to that of other species were identified (*Rubrobacter xylanophilus; Candidatus Amoebophilus asiaticus; Stenotrophomonas maltophilia; Ishikawaella* symbiont of *Megacopta cribraria*); their possible role in the interaction with viral coat proteins is not yet known. Thus, the array of possible interactions between different GroELs and viruses remains to be deciphered.

### 2.3. The Worldwide Spread of TYLCV

Among the begomoviruses transmitted, some may cause plant diseases of considerable economic and social importance. Tomato yellow leaf curl disease (TYLCD) is one of the most devastating plant diseases in warm and temperate regions of the world [101,102]. TYLCD infections result in yellowing and upwards curling of leaflet margins, stunting of the plants and flower abortion. Infected plants are less vigorous and produce fruits with reduced market value. Moreover, infection during early growth stages can lead to total loss of the crop [103,104].

The first description of TYLCD-like symptoms dates back to the late 1920s in the Jordan Valley in Israel (cited in [105]). Severe outbreaks of the disease occurred in the early 1960s in Israel [105]. From the late 1980s on, a major and rapid geographic spread of TYLCD took place and its distribution area now stretches from Japan in the east to Spain in the west in the Old World, and to Reunion Island and Australia in the south [105]. In the 1990s, TYLCD reached the New World and rapidly spread in North America as well as in the Caribbean [106,107,108].

It was not until 1991 that the first molecular data on the viral agent associated with TYLCD became available and was named *Tomato yellow leaf curl virus*-Israel (TYLCV-IL; e.g. TYLCV-IL[IL:Reo:86]; [109,110]). Subsequently, another begomovirus strain associated with TYLCD infections was cloned and named *Tomato yellow leaf curl virus*-Mild (TYLCV-Mld; TYLCV-Mld[IL:93]) [111]. Ever since, an increasing number of tomato yellow leaf curl genomes have been sequenced and molecularly characterized revealing the wide viral diversity associated with TYLCD [20]. Based on phylogenetic relationships, there are currently six related species and 15 strains, referred to as TYLCV-like viruses, associated with TYLCD: TYLCV, *Tomato yellow leaf curl Sardinia virus* (TYLCSV), *Tomato yellow leaf curl Axiarqua virus* (TYLCAxV), *Tomato yellow leaf curl Malaga virus* (TYLCMalV), *Tomato yellow leaf curl Mali virus* (TYLCMV) and *Tomato leaf curl Sudan virus* (ToLCSDV) [112]. Among them, TYLCV, the best known and emergent species, have the broadest geographical range. Based on sequence identities, five strains of TYLCV are currently described including Gezira (e.g., TYLCV-Gez [SD:96]), Iran (e.g., TYLCV-IR [IR:Ira:98]), Israel (e.g., TYLCV-IL [IL:Reo:86]), Mild (e.g., TYLCV-Mld [IL:93]) and Oman strains (e.g., TYLCV-OM [OM:Alb:05]).

**Figure 1:**
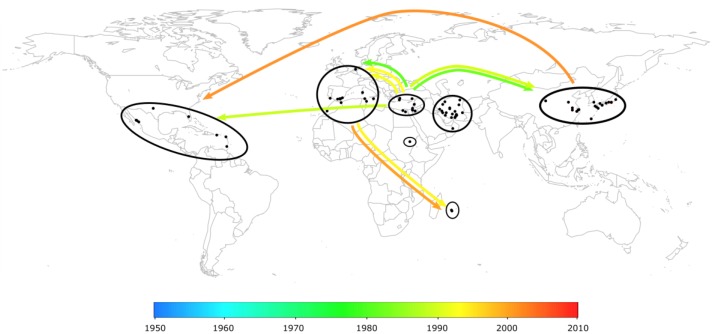
Worldwide spread of *Tomato yellow leaf curl virus* (TYLCV) inferred using phylogeography analyses on the coat protein and full genome datasets [114]. According to the color scale at the bottom of the figure, arrow colors depict the mean ages (in years) of the migration events.

Because of the great TYLCV diversity encountered in Mediterranean basin and the Middle East, this region was suspected to be the center of origin of these viruses [113]. Using Bayesian phylogeopraphic inference and recombination analyses, Lefeuvre *et al.* [114] recently reconstructed a plausible history of the diversification and movement of TYLCV throughout the world (Figure 1). In accordance with historical records, this study suggests that the first TYLCV most probably arose in the Middle East between the 1930s and 1950s and that the global spread of TYLCVs only began in the 1980s after the emergence of TYLCV-Mld and TYLCV-IL. Evidence was also provided that the highly invasive TYLCV-IL has jumped at least twice to the New World, once from the Mediterranean basin in the 1990s, and once from Asia in the 2000s. This study also identified the Middle East and the regions surrounding Iran in particular as the most probable current and past centers of ongoing TYLCV diversification. Nevertheless, although this region displays intensive TYLCV diversification, it was also found to be epidemiologically isolated, suggesting that the novel variants found there may never disseminate outside the Middle East. In contrast, the Mediterranean basin was identified as the main launch-pad of global TYLCV movements and the direct source of the TYLCV variants currently spreading around the world.

It is important to note that this pattern of invasion resembles the recently described phenomenon of the invasive bridgehead effect [115]. In this scheme, widespread invasions do not result from isolates originating from across the native range of the invasive species but rather from individual particularly invasive sub-populations that serve as sources of colonists for the conquest of new territories. While the invasive bridgehead effect is evolutionarily parsimonious [115,116], data and examples remain too scarce to investigate whether this pattern of invasion is a common mechanism, and it would thus be of particular interest to explore genetic data sets from other worldwide invasive species.

### 2.4. Case Study in Reunion Island

Reunion Island is a French island situated in the Mascarenes archipelago, in the south-western part of the Indian Ocean (700 km east of Madagascar). As colonization and the development of agriculture in Reunion Island are recent, imprints of biological invasion are particularly easy to track and make the island an ideal venue to study biological invasions.

#### 2.4.1. Invasion of the World Invasive MEAM1 Species of *Bemisia Tabaci*

**Figure 2:**
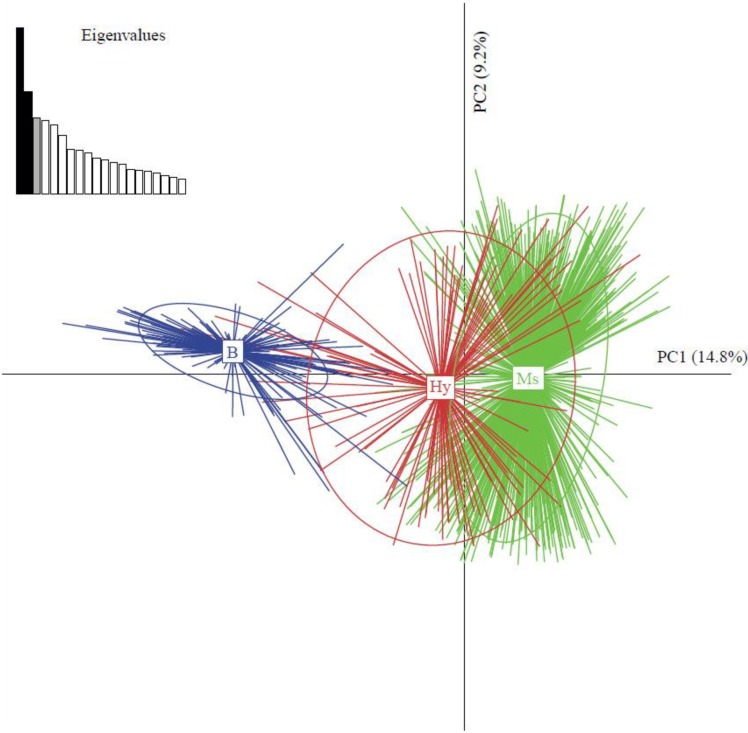
Multivariate analysis of *Bemisia tabaci* populations in Reunion Island [72]. First and second components of a principal component analysis of 10-loci microsatellites from *B. tabaci* females from the three populations MEAM1 (biotype B), Hybrids (Hy) and Indian Ocean (biotype Ms) defined by STRUCTURE analysis. The relative contributions of the two first axes (PC1 and PC2) to the total genetic variation are 14.8% and 9.2% respectively. Eigenvalues corresponding to the represented components are in black. Populations are labeled inside their 95% inertia ellipses.

*B. tabaci* was first described in Reunion Island in 1938 [117] and later by Luziau [118]. After its introduction in the late 1990s, the world invasive MEAM1 species of *B. tabaci* became a major threat to tomato crops as a vector of TYLCV [99,119,120,121]. RAPD-PCR and *COI* sequencing on *B. tabaci* populations of this island also revealed the presence of a new undescribed species, named Ms (Indian Ocean putative species, according to Dinsdale *et al*. [33]), indigenous to the south-western Indian Ocean [122,123]. The genetic diversity of these two putative species was later analyzed using microsatellite markers on field samples from 2001 and 2002, and wild hybrids of multiple generation between MEAM1 and Indian Ocean species were subsequently characterized [72,123]. In 2006, samples were collected at the same sites as in 2001 and 2002 and genetic analyses using principle component analyses (PCA, Figure 2) and a Bayesian clustering method (STRUCTURE) confirmed the presence of admixed individuals of multiple generations between MEAM1 and Indian Ocean putative species.

#### 2.4.2. Co-Existence of Invasive and Indigenous Species and Hybrids of *Bemisia Tabaci*

**Figure 3:**
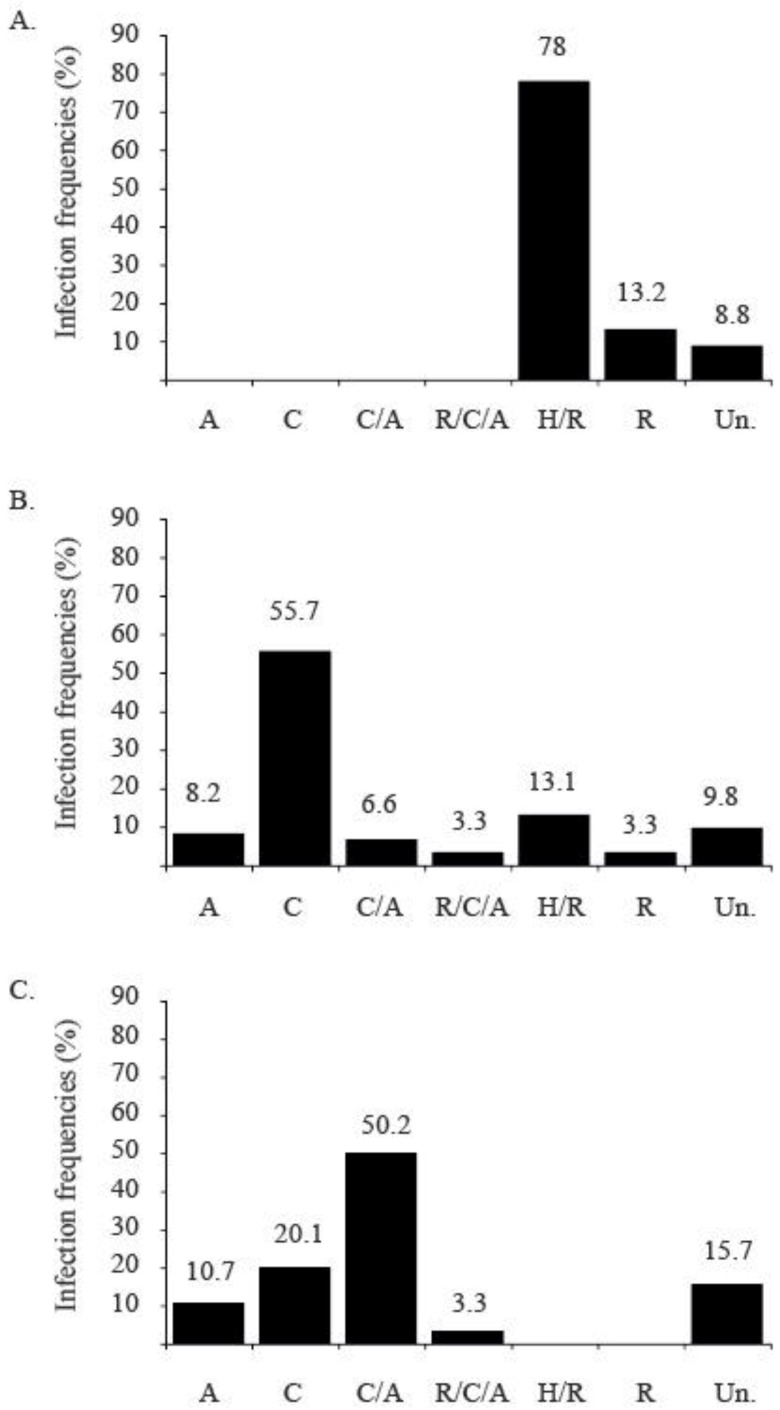
Infection frequencies of the different endosymbiont combinations observed in individuals of MEAM1 (**A**), Hybrids (**B**) and Indian Ocean (**C**) populations of *Bemisia tabaci* collected in the field in Reunion Island. A: *Arsenophonus*, C: *Cardinium*, H: *Hamiltonella*, R: *Rickettsia*, and Un.: Undetected. [72].

More than 12 years of intensive field surveys after the introduction of MEAM1 species demonstrated that the indigenous species is still present in Reunion Island (unpublished data), although in other countries, MEAM1 or MED have displaced indigenous populations [32,124]. The limited success of MEAM1 in Reunion Island may be explained by a combination of several factors. Firstly, the endosymbiont populations of the different *B. tabaci* populations, which have been characterized in MEAM1 and Indian Ocean species (and the hybrids of these species; [72]) may hold some clues. MEAM1 harbors *Rickettsia* and *Hamiltonella* as in other world populations, and Indian Ocean harbours *Cardinium* and *Arsenophonus*. Endosymbiont analysis of the hybrid population indicated that matings involving Indian Ocean females and MEAM1 males were more successful than reciprocal matings (Figure 3). These results may partly explain why MEAM1 has not yet displaced the Indian Ocean species in Reunion Island. Secondly, coexistence of invasive and indigenous *B. tabaci* species might be facilitated by spatial and temporal niche partitioning [56]. In Reunion Island, Delatte *et al.* [123] demonstrated that MEAM1 and Indian Ocean coexist in sympatry throughout most of their geographical ranges, although they tend to segregate into different types of host plants with MEAM1 predominating in vegetable crops, and Indian Ocean being more frequently found on weeds. Endosymbionts could enhance postzygotic barriers involved in non-random hybridization between MEAM1 and Indian Ocean [72] and niche partitioning implicated in prezygotic barriers could explain the coexistence of an invasive and indigenous species.

Most interestingly, analyses of samples in Reunion Island in 2010 indicated the additional presence of MED [124]. The interactions between MEAM1 and Indian Ocean putative species, monitored in the field since 1997, will thus be complemented from 2010 onwards by MED as a new actor.

#### 2.4.3. Successive Introduction of TYLCV Strains

Although indigenous populations of *B. tabaci* were detected in Reunion Island, no indigenous begomoviruses infecting tomato were described before the introduction of TYLCV-Mld in 1997. Following its first description in the southern part of the island, TYLCV-Mld was detected in almost all the tomato-growing area by the end of 1998. With severe economic losses observed (up to 85%) in outdoor and/or protected tomato crops, TYLCV became the main limiting factor to tomato production [119,120,125].

This accidental introduction in a remote agroecosystem, apparently free of any other tomato-infecting begomoviruses, provided an opportunity to study the molecular evolution of TYLCV-Mld from the initial inoculum. The genetic variation of TYLCV-Mld was monitored in a field survey conducted between 1997 and 2004 in the main tomato growing areas. The very low diversity of the isolates in 1997 did not provide any evidence of multiple TYLCV introductions. A quasi-linear increase in genetic diversity was observed in the following years. In addition, the population effective size of TYLCV-Mld underwent a sudden increase from 2001 to 2004, consistent with a founder effect due to the introduction of a small number of virus individuals [126].

In 2004, particularly severe symptoms of TYLCD were observed in the western part of the tomato cropping region. Molecular diagnosis revealed the introduction of the “Israel” strain also called “the severe” strain of TYLCV (TYLCV-IL) [127]. This introduction provided an ideal model to study the competition between two strains of one of the most emergent virus species. While studies describing epidemiological factors (*i.e.*, host range, vector transmission efficiencies) associated with TYLCD epidemics have been performed in temperate regions, extensive studies in tropical insular ecosystems are still rare. Different scenarios can be expected in such an epidemiological situation with competition: synergism, co-existence, displacement, or emergence of new variants. Studies describing epidemiological dynamics of TYLCV-like viruses in Spain or Italy revealed the displacement of one species by the other [128,129], the co-existence of the two species [129] or the emergence of a new severe recombinant variant [130,131,132]. After a seven-year period following its emergence in Reunion Island, TYLCV-IL has progressively displaced TYLCV-Mld, which is now predominantly detected in mixed infections [133]. The characterization of the key biological traits involved in this epidemiological pattern is underway [134] and should reveal clues to virus competition that may help predict the outcome of possible future introductions.

## 3. Major Conclusions and Perspectives

Invasions of arthropod vectors and viruses are the main factors associated with viral emerging diseases. This is particularly the case for begomoviruses transmitted by *B. tabaci*, which are responsible for the emergence of one of the most devastating plant diseases in the world (TYLCD). The first key factor identified in the worldwide dissemination of TYLCD is the worldwide expansion of invasive, polyphagous and in some cases pesticide resistant species (MEAM1 or MED) of *B. tabaci* [32,38,55]. It has also become clear that endosymbionts play a fundamental role in *B. tabaci* host ecology and evolution through a wide range of effects, such as increasing host survival or fecundity. Furthermore, the introduction of invasive species of *B. tabaci* into new ecosystems has provided opportunities for several begomoviruses to jump from natural flora to contaminate crops such as tomato. This phenomenon, recently named the weed-crop connection [31], is illustrated by the recent emergence and wide range of begomoviruses involved in viral epidemics on tomato in Brazil since the mid-1990s [31,135,136]. The second key factor is the introduction of plant viruses in a new area. This is the case for the worldwide spread of TYLCV invasive strains (TYLCV-IL and -Mld). In agreement with historical records, Lefeuvre *et al.* [114] recently suggested that the first TYLCVs most probably arose in the Middle East between the 1930s and 1950s, and that the global spread of TYLCVs only began in the 1980s after the emergence of TYLCV invasive strains. Moreover, the Mediterranean basin was identified as the main launch-pad of global TYLCV movements and the direct source of the TYLCV variants currently spreading around the world. This pattern of invasion is similar to the recently described phenomenon of the invasive bridgehead effect where a particularly invasive population serves as source of colonists for the conquest of new territories [115]. Recent studies have demonstrated that recombination plays a major role in the evolution and emergence of begomoviruses (for review see [1,137,138]). This is apparently the case of the invasive IL strain of TYLCV, which is a natural recombinant which arose by recombination between TYLCV-Mld and Tomato leaf curl virus (ToLCV)-like ancestors [139].

The introduction of plant viruses in a new area is mainly triggered by long distance exchange of plant material or vector insects due to the global trade in vegetables and ornamental plants [140]. A well-documented case of the importance of international trade causing long-distance dispersal of viruses was the original introduction of TYLCV-IL into the New World (Florida) from the Old World in 1992 via infected transplants that were probably purchased in Israel [141]. This example also illustrates the great capacity of the invasive TYLCV to very rapidly adapt to the new niches it invades; in some cases even causing the disappearance of indigenous begomovirus species. In the case of Reunion Island, the successive arrivals of TYLCV-Mld and MEAM1 (1997), TYLCV-IL (2004) and MED (2010) provide a wide range of possible interactions with the indigenous Indian Ocean species of *B. tabaci* in the field. The future of the interaction between the three species of *B. tabaci* and the competition between invasive strains of TYLCV in the fields of Reunion Island is a fortuitous experiment that is currently underway.
